# Anthropometric and blood parameters for the prediction of NAFLD among overweight and obese adults

**DOI:** 10.1186/s12876-018-0840-9

**Published:** 2018-07-13

**Authors:** Tilman Kühn, Tobias Nonnenmacher, Disorn Sookthai, Ruth Schübel, Daniel Antonio Quintana Pacheco, Oyunbileg von Stackelberg, Mirja E. Graf, Theron Johnson, Christopher L. Schlett, Romy Kirsten, Cornelia M. Ulrich, Rudolf Kaaks, Hans-Ulrich Kauczor, Johanna Nattenmüller

**Affiliations:** 1 German Cancer Research Center (DKFZ), Division of Cancer Epidemiology, Im Neuenheimer Feld 581, 69120 Heidelberg, Germany; 2 Department of Diagnostic and Interventional Radiology, University Hospital Heidelberg, Im Neuenheimer Feld 110, D-69120 Heidelberg, Germany; 3 National Center for Tumor Diseases (NCT), Liquid Biobank, Im Neuenheimer Feld 460, 69120 Heidelberg, Germany; 4 Division of Preventive Oncology, German Cancer Research Center (DKFZ) and National Center for Tumor Diseases (NCT), Im Neuenheimer Feld 460, 69120 Heidelberg, Germany; 5 Department of Population Health Sciences, University of Utah, 2000 Circle of Hope, Salt Lake City, UT 84112-5550 USA; 6 Huntsman Cancer Institute, Salt Lake City, 2000 Circle of Hope, Salt Lake City, UT 84112-5550 USA

**Keywords:** Non-alcoholic fatty liver disease (NAFLD), Magnetic resonance images (MRI), Liver fat content, Obesity, ALT, Insulin

## Abstract

**Backround:**

Non-alcoholic fatty liver disease (NAFLD) comprises non-progressive steatosis and non-alcoholic steatohepatitis (NASH), the latter of which may cause cirrhosis and hepatocellular carcinoma (HCC). As NAFLD detection is imperative for the prevention of its complications, we evaluated whether a combination of blood-based biomarkers and anthropometric parameters can be used to predict NAFLD among overweight and obese adults.

**Methods:**

143 overweight or obese non-smokers free of diabetes (50% women, age: 35–65 years) were recruited. Anthropometric indices and routine biomarkers of metabolism and liver function were measured to predict magnetic resonance (MR) - derived NAFLD by multivariable logistic regression models. In addition, we evaluated to which degree the use of more novel biomarkers (adiponectin, leptin, resistin, C-reactive protein, TNF-α, IL-6, IL-8 and interferon-γ) could improve prediction models.

**Results:**

NAFLD was best predicted by a combination of age, sex, waist circumference, ALT, HbA1c, and HOMA-IR at an area under the receiver operating characteristic curve (AUROC) of 0.87 (95% CI: 0.81, 0.93) before and 0.85 (95% CI: 0.78, 0.91) after internal bootstrap validation. The use of additional biomarkers of inflammation and metabolism did not improve NAFLD prediction. Previously published indices predicted NAFLD at AUROCs between 0.71 and 0.82.

**Conclusions:**

The AUROC of > 0.8 obtained by our regression model suggests the feasibility of a non-invasive detection of NAFLD by anthropometry and circulating biomarkers, even though further increments in the capacity of prediction models may be needed before NAFLD indices can be applied in routine clinical practice.

**Electronic supplementary material:**

The online version of this article (10.1186/s12876-018-0840-9) contains supplementary material, which is available to authorized users.

## Backround

Non-alcoholic fatty liver disease (NAFLD), i.e. the accumulation of liver fat in individuals without excessive alcohol consumption, use of steatogenic medication or strong genetic predisposition, is highly prevalent in many areas of the world [1, 2]. For example, representative data from the United States indicate that 19% of Americans may have NAFLD, and similar prevalence rates have been reported from several regions in Asia [1, 3]. Considering the strong positive association between obesity and NAFLD, it can be expected that the incidence of NAFLD will rise with increasing obesity rates [1, 4].

NAFLD comprises non-progressive steatosis and non-alcoholic steatohepatitis (NASH), the latter of which may cause cirrhosis and hepatocellular carcinoma (HCC). Moreover, NAFLD is closely linked to subclinical inflammation and insulin resistance, and has been considered as the hepatic manifestation of the metabolic syndrome [1, 2]. In fact, it has been proposed that NAFLD may be a major risk factor not only for cirrhosis and HCC, but also cardiovascular disease (CVD) and extra-hepatic cancers driven by an unfavorable metabolic risk profile [4, 5].

Given the high prevalence of NAFLD and its possible severe health consequences, its detection before progression into steatohepatitis, advanced fibrosis and cirrhosis is imperative, particularly because steatosis alone is rapidly reversible through lifestyle modification and particularly through weight loss [6, 7]. At the same time, routine detection of NAFLD based on liver biopsy, sonography and imaging may not be feasible with respect to health care expenditures and biopsy-related risks [8]. Thus, we tested the performance of published algorithms based on combinations of anthropometric indices and routine blood biomarkers [9–13] in the prediction of MRI-derived liver fat content and NAFLD. Analyses were carried out in a sample of overweight and obese non-diabetics, i.e. a potentially ideal target population for NAFLD prevention. Moreover, we examined if additional biomarkers that have been proposed in the literature may improve the prediction of liver fat content and NAFLD. Our goal was to assess whether combinations of anthropometric parameters and circulating biomarkers can serve as an efficient pre-screening tool for the detection of NAFLD and steatohepatitis in primary care, upstream of sonography-, imaging-, and biopsy-based diagnostic tests.

## Methods

### Study population

We evaluated baseline data of the HELENA Trial, a randomized dietary intervention study that has been described in detail elsewhere [14]. In brief, 150 non-diabetic overweight and obese non-smokers (50% females) aged 35–65 years entered the study between May 2015 and May 2016. Participants were randomly assigned to three groups (intermittent calorie restriction, continuous calorie restriction, control group) for a 12-week intervention phase, a 12-week follow-up phase and a 26 week follow-up phase. The main objective of the trial was to evaluate whether intermittent calorie restriction has stronger effects on metabolic parameters, body composition, and psychosocial factors than continuous calorie restriction.

At screening, study physicians carried out a general medical assessment including anthropometric measurements and a blood draw. The following exclusion criteria were applied: Prevalent diabetes mellitus or HbA1c levels ≥6.5% or fasting plasma glucose levels > 126 mg/dl; known liver dysfunction or increased GGT (women: > 60 U/l; men: > 80 U/l), AST (> 40 U/l; > 50 U/l), or ALT (> 50 U/l; > 65 U/l) levels; known kidney dysfunction or increased urea (> 50 mg/dl; > 50 mg/dl), uric acid (> 8 mg/dl; > 9 mg/dl) or creatinine (> 1 mg/dl, > 1.3 mg/dl), known thyroid dysfunction or increased (> 4.4 mU/l; > 4.4 mU/l) or decreased (< 0.36 mU/l; < 0.36 mU/l) thyroid-stimulating hormone (TSH) levels; history of cancer within the past ten years; history of eating disorders (e.g. bulimia, binge-eating); severe bleeding tendency; use of medication for immunosuppression or modulation of fat metabolism; participation in an intervention study within the past three months; use of hormone replacement therapy; pregnancy or breastfeeding within the past 12 months. Further exclusion criteria covered typical MRI-contraindications (i.e., claustrophobia, cardiac pacemakers or defibrillators, non-removable electronic implants or devices, non 1.5 Tesla-MRI approved medical foreign bodies, implants and orthopedic foreign bodies, joint end prostheses, or other metallic foreign bodies). Alcohol consumption was assessed by 7-day dietary records, which were analyzed by PRODI 6.3 (Nutri-Science GmbH, Hausach, Germany).

The study was carried out at the German Cancer Research Center (DKFZ), Heidelberg, Germany and the Heidelberg University Hospital (imaging component, see below). It was registered at ClinicalTrials.gov under NCT02449148. The guidelines of the Helsinki Declaration were applied and the study was approved by the Ethics Committee of the Heidelberg University Hospital (Heidelberg, Germany). All participants provided written informed consent.

### Laboratory methods

Clinical biochemistry markers (ALT, AST, GGT, ALP, HDL-cholesterol, total cholesterol, triglycerides, fasting glucose, HbA1c, albumin, bilirubin, ferritin, transferrin, total iron binding capacity) were measured directly after blood draw at the Central Laboratory, Heidelberg University Hospital by routine assays. Remaining samples were processed into EDTA-plasma, serum and buffy coat, and frozen at − 80 °C. Plasma levels of adiponectin, leptin, resistin, insulin, C-reactive protein (CRP), tumor necrosis factor-α (TNF-α), interleukin-6 (IL-6), interleukin-8 (IL-8) and interferon-γ (IFN-γ) were measured in the laboratory of the Division of Cancer Epidemiology at DKFZ. A single aliquot was thawed immediately prior to the analyses in January 2017 without previous or further freeze thaw cycles. All analytes were measured by electrochemiluminescence immunoassays (ECLIA) on the “Quickplex SQ 120” instrument from Meso Scale Discoveries (MSD, Maryland, USA), using singleplex (insulin, leptin, CRP, IL-8, resistin, and adiponectin) and multiplex (TNF-α, IL-6, and IFN-γ) kits from MSD.

### Imaging

Liver fat content was measured using a multi-echo GRE technique [15, 16] (Siemens LiverLab, Siemens Healthcare, Erlangen, Germany) during a 15 min MRI examination at the Department for Diagnostic and Interventional Radiology, University Hospital Heidelberg. A 1.5 Tesla MRI scanner with a 70 cm bore design (MAGNETOM Aera; Siemens Healthcare, Erlangen, Germany) was used. Hardware, MR-protocol and software remained constant between all MRI-scans. Details on the protocol have previously been described [14, 15].

**Fig. 1:**
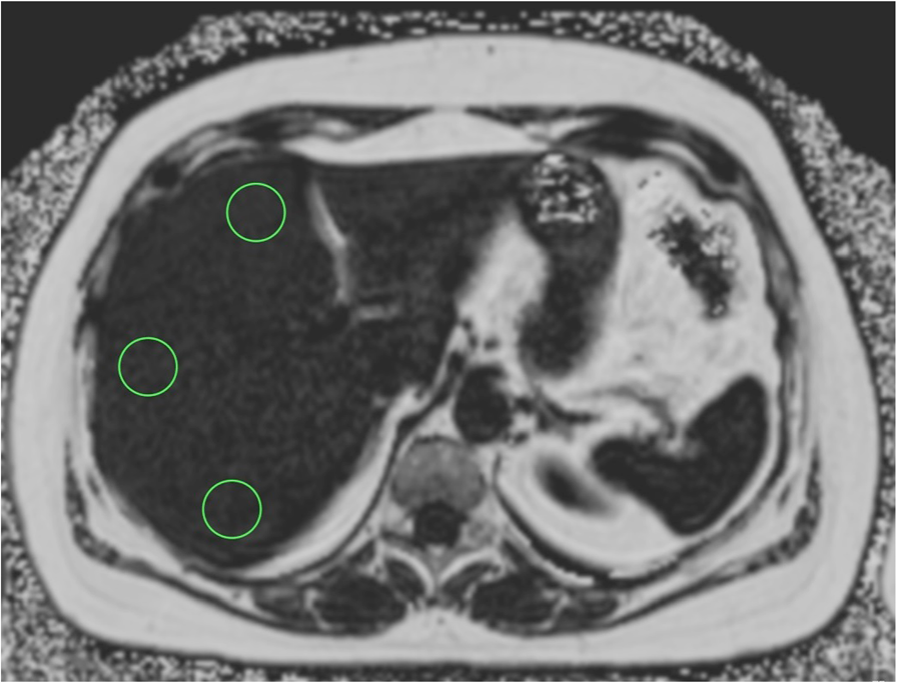
MR-image of liver with positioning of three identical regions of interest in liver segment 4,7 and 8 using the proton density fat fraction (PDFF) map, area of each ROI 4.00 cm^2^, with the unit of one gray value corresponding to a fat content of 0.1% per voxel

Imaging data on liver fat content was evaluated on a post-processing software (OsiriX, Pixmeo SARL, Bernex, Switzerland) manually by one reader (T.N.) using the proton density fat fraction (PDFF) map, based on mean counts from three identical regions of interest (ROI, each area 4.00cm^2^) positioned dorsally, anterior-medially and anterior-laterally of the right liver lobe at a level immediate cranial of the liver hilum, with the unit of one gray value corresponding to a fat content of 0.1% per voxel [16] (see Fig. 1). For the ROI placement, larger vessels and connective tissue was avoided. ROI-data were exported using a XML-format and could be accessed for further statistical analysis. Results were validated by a second reader (J.N.). Intra- and Inter-rater coefficients of correlation were 0.99 and 0.99, respectively, indicating excellent reliability of our liver fat quantification.

### Statistical analyses

Characteristics of the study population are shown as arithmetic mean values (continuous variables) and proportions (categorical variables). To analyze the correlations between anthropometric parameters, blood biochemistry markers, and liver fat content, Spearman’s coefficients were calculated. Moreover, multivariable linear regression analyses were used to establish a prediction model for liver fat content on the continuous scale. Predictors of liver fat content were selected by backward elimination at a *p*-value threshold of 0.05 based on 100 bootstrap samples entering the biomarkers described above and all anthropometric parameters (height, weight, BMI, waist circumference and hip circumference) together into the model. Predictors were chosen in case of significant associations in two thirds of the bootstrapped multivariable regression analyses. Age and sex were forced into the multivariable model.

For the prediction of NAFLD as a dichotomous outcome, we classified liver fat values > 5.56% as indicative of NAFLD, as proposed by Szczepaniak et al. [17]. Again, backward elimination at a p-value threshold of 0.05 based was applied to determine the final set of predictors, this time using multivariable logistic regression models, again forcing age and sex into the models, and entering all available biomarkers and anthropometric parameters. The predictive power of the final combination of variables was evaluated by areas under the receiver-operating characteristic curves (AUROC). For internal validation, logistic regression analyses were corrected for optimism using average beta estimates from 100 bootstraps.

In addition to our own prediction models, we tested the following four published algorithms to predict NAFLD:

- NAFLD liver fat score [9]:

$$ \mathrm{NAFLD}\ \mathrm{liver}\ \mathrm{fat}\ \mathrm{score}=-2.89+{1.18}^{\ast }\ \mathrm{metabolic}\ \mathrm{syndrome}\ \left(\mathrm{yes}=1,\mathrm{no}=0\right)+{0.45}^{\ast }\ \mathrm{type}\ 2\ \mathrm{diabetes}\ \left(\mathrm{yes}=1,\mathrm{no}=0\right)+{0.15}^{\ast }\ \mathrm{fS}-\mathrm{insulin}\ \left(\mathrm{mU}/\mathrm{L}\right)+{0.04}^{\ast }\ \mathrm{fS}-\mathrm{AST}\ \left(\mathrm{U}/\mathrm{L}\right)-{0.94}^{\ast }\ \mathrm{AST}/\mathrm{ALT} $$

b) Framingham Steatosis Index (FSI) [10]:

$$ \mathrm{FSI}=-7.981+{0.011}^{\ast }\ \mathrm{age}\ \left(\mathrm{years}\right)-{0.146}^{\ast }\ \mathrm{sex}\ \left(\mathrm{female}=1,\mathrm{male}=0\right)+0.173\ \mathrm{BMI}\ \left(\mathrm{kg}/{\mathrm{m}}^2\right)+{0.007}^{\ast }\ \mathrm{triglycerides}\ \left(\mathrm{mg}/\mathrm{dL}\right)+{0.593}^{\ast }\ \mathrm{hypertension}\ \left(\mathrm{yes}=1,\mathrm{no}=0\right)+{0.789}^{\ast }\ \mathrm{diabetes}\ \left(\mathrm{yes}=1,\mathrm{no}=0\right)+{1.1}^{\ast }\ \mathrm{ALT}/\mathrm{AST}\ \mathrm{ratio}\ge 1.33\ \left(\mathrm{yes}=1,\mathrm{no}=0\right) $$

c) The Hepatic Steatosis Index (HSI) [11]:

$$ HSI={8}^{\ast }\  ALT/ AST\  ratio+ BMI\ \left(+2, if\  DM;+2, if\ female\right) $$

d) The Fatty Liver Index (FLI) [12]:

$$ FLI=\left({e}^{0.953\ast loge\ (triglycerides)+0.139\ast BMI+0.718\ast loge\ (ggt)+0.053\ast waist\ circumference-15.745}\right)/{\left(1+{e}^{0.953\ast loge\ (triglycerides)+0.139\ast BMI+0.718\ast loge\ (ggt)+0.053\ast waist\ circumference-15.745}\right)}^{\ast}\;100 $$

We further applied the following formula developed by Kotronen et al. [9] to predict liver fat content (%):

$$ Liver\  fat\ \left(\%\right)={10}^{\left(-0.805+0.282\ast metabolic\ syndrome\ \left( yes=1, no=0\right)+0.078\ast type\ 2\  diabetes\ \left( yes=1, no=0\right)+0.525\  LOG\right( fS- insulin\ \left( mU/L\right)+0.521\ast LOG\Big( fS- AST\ \left(U/L\right)-0.454\ast LOG\left( AST/ ALT\right)} $$

## Results

### Characteristics of the study population

Out of the 150 participants in the HELENA-Trial, 145 gave written informed consent for the MRI examination. One stopped the MRI examination due to claustrophobia, and there was a technical failure regarding liver fat quantification in another case. Thus, liver fat values were available for 143 individuals with a mean age of 50.1 ± 8.1 years, and a mean BMI of 31.4 ± 3.7 (see Table 1). Participants showed an average liver fat content of 7.7%, and the prevalence of NAFLD was 52.5% (see Table 2). The majority of the study participants had liver fat contents below 15%, and only one participant had a liver fat content > 30% (see Additional file 1: Figure S1).

**Table 1:** Characteristics of the study population (*n* = 143)

*General characteristics*	
Age	50.1 ± 8.1
Women (%)	49.7
University Degree (%)	46.1
Alcohol intake (g/d)^a^	11.0 ± 13.3
*Anthropometry*	
BMI (kg/m^2^)	31.4 ± 3.7
Waist circumference (women) (cm)	99.5 ± 10.1
Waist circumference (men) (cm)	109.1 ± 10.6
Height (cm)	172.9 ± 9.8
*Liver fat*	
Liver fat content (%)	7.7 ± 6.0
NAFLD (%)^b^	52.5
*Liver function*	
AST (U/l)	22.9 ± 5.3
ALT (U/l)	26.7 ± 11.1
AST to ALT ratio	1.0 ± 0.3
GGT (U/l)	26.8 ± 15.8
ALP (U/l)	70.7 ± 16.9
LDH (U/l)	196.6 ± 28.7
Albumin (g/l)	43.7 ± 2.2
Bilirubin (mg/dl)	0.7 ± 0.3
*Kidney function*	
Uric acid (mg/dl)	30.1 ± 6.8
Creatinine (mg/dl)	0.8 ± 0.1
*Lipid metabolism*	
HDL (mg/dl)	54.0 ± 14.2
LDL (mg/dl)	125.9 ± 27.1
Triglycerides (mg/dl)	132.8 ± 80.7
*Glucose metabolism*	
Glucose (nmol/l)	5.2 ± 0.4
HbA1c (%)	5.5 ± 0.3
Insulin (pg/ml)	6.8 ± 4.3
HOMA-IR (μU/mmol/l)	1.6 ± 1.1
*Iron metabolism*	
Ferritin (μg/l)	148.8 ± 142.0
Iron (μmol/l)	17.9 ± 6.6
Total Iron Binding Capacity (μmol/l)	61.6 ± 7.7
Transferrin (g/l)	2.9 ± 0.4
*Adipokines and cytokines*	
CRP (pg/ml)	3.1 ± 3.3
Adiponectin (pg/ml)	17.5 ± 11.2
Leptin (pg/ml)	8.9 ± 9.4
Resistin (pg/ml)	4.3 ± 1.8
IL-6 (pg/ml)	1.6 ± 2.0
IL-8 (pg/ml)	11.1 ± 12.3
Interferon-γ (pg/ml)	14.8 ± 13.9
TNF-α (pg/ml)	4.5 ± 2.6

Continuous values shown as arithmetic mean ± standard deviation;

^a^as assessed by 7-day dietary record

^b^Non-alcoholic fatty liver disease (liver fat content > 5.56%)

**Table 2:** Proportion of individuals with NAFLD^a^

Overall	52.5%
Women	43.6%
Men	61.1%
BMI: 25–29.9^b^	35.7%
BMI: 30–34.9^b^	62.3%
BMI: 35–39.9^b^	65.4%

^a^Non-alcoholic fatty liver disease (liver fat content > 5.56%)

^b^Proportions of individuals with BMI values of 25–29.9, 30–34.9, and 35–39.9 were: 39.2 (%), 42.7 (%) and 18.2 (%)

### Correlates of liver fat

Correlations between liver fat content (%) and the following parameters were observed at a Spearman’s |ρ| > 0.3 and *p*-values < 0.001: BMI (0.33), waist circumference (0.52), ALT (0.56), AST/ALT ratio (− 0.54), HDL (− 0.36), triglycerides (0.33), ferritin (0.32), HbA1c (0.31), Insulin (0.55), and HOMA-IR (0.56) (see Additional file 1: Figure S2). By contrast, there was no significant correlation between self-reported alcohol intake and liver fat content (ρ = 0.08).

After backward selection, waist, ALT, GGT, HbA1c, insulin, and creatinine were significantly associated with liver fat content in the multivariable linear regression model at p-values < 0.05. The overall R^2^ of the model after internal bootstrap validation was 53.9%. The strongest predictor of liver fat content was ALT, with a semi-partial R^2^ of 30.1%, followed by HbA1c (10.6%), and GGT (6.2%) (Table 3). None of the other parameters explained more than 5% of the variance in liver fat content. Applying the score developed by Kotronen et al. [9] to predict liver fat content our multivariable linear regression analysis revealed a model R^2^ of 41.4%.

**Table 3:** Predictors of liver fat content (%)^a^

	Semi-partial R^2^ (Type II)^b^	*p*
Age	< 1%	0.88
Sex	3.5%	0.03
Waist	4.9%	< 0.01
ALT	30.1%	< 0.01
GGT	6.2%	< 0.01
HbA1c	10.6%	< 0.01
Insulin	3.8%	0.02
Creatinine	3.5%	0.03

^a^Overall R^2^: 53.9%

^b^Do not add up to overall R^2^

### NAFLD prediction models

**Fig. 2:**
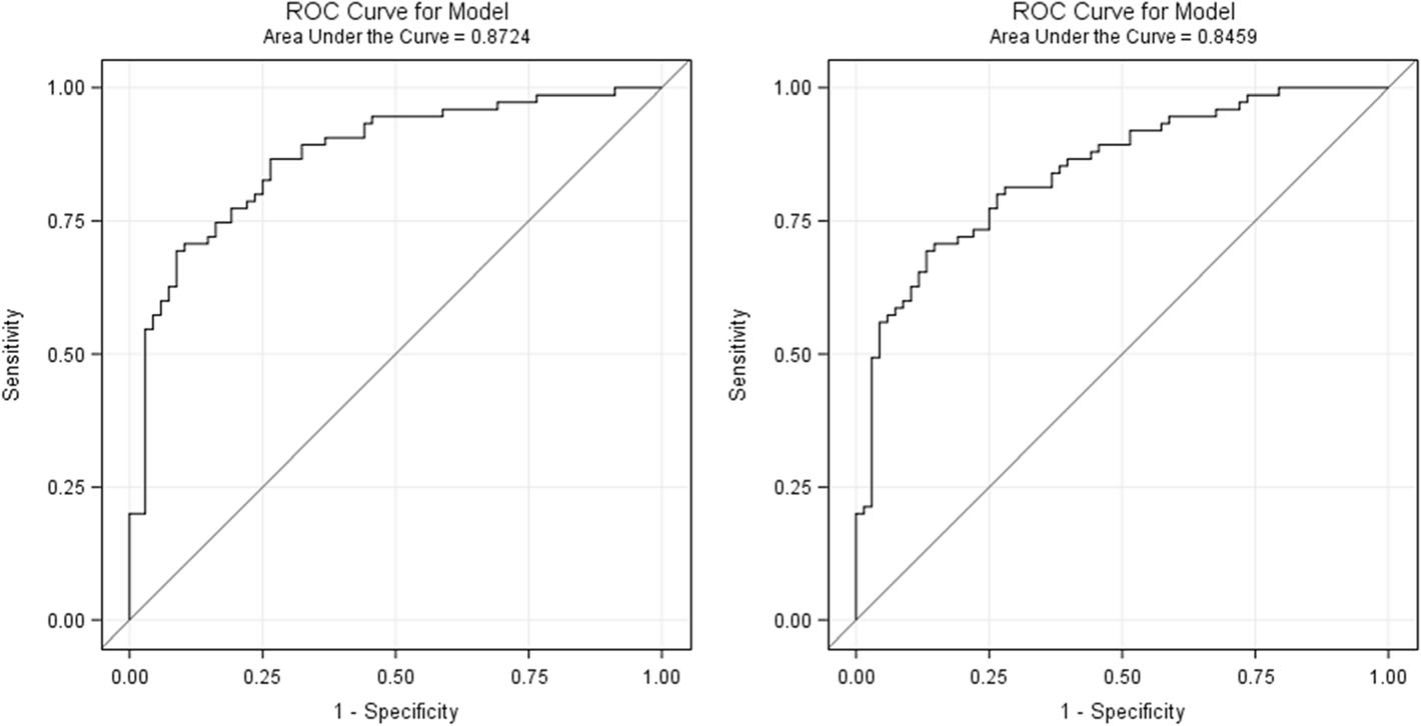
ROC curves for NAFLD prediction models from logistic regression analyses before (left) and after (right) internal bootstrap validation. Our validated model has the following regression formula: NAFLD = − 22.8113 + 0.00317*Age - 0.5036*Female Sex + 0.0609*Waist (cm) + 0.1292 ALT (U/l) + 2.1868*HbA1c (%) + 0.8066*HOMA-IR (μU*/mmol/l)*

Associations between individual predictors and the odds ratio of NAFLD are shown in Additional file 1: Table S1. The strongest associations were observed for ALT, HOMA-IR, Insulin, and waist circumference, with areas under the ROC curve of 0.78, 0.76, 0.76, and 0.75. Our final multivariable logistic regression model to predict NAFLD included age, sex, waist circumference, ALT, HbA1c, and HOMA-IR. The area under the ROC curve was 0.87 (95% CI: 0.81, 0.93) before and 0.85 (95% CI: 0.78, 0.91) after internal bootstrap validation (see Fig. 2). At a sensitivity of 100% and a specificity of 0% (no false negatives), our validated prediction model would have revealed a wrong “diagnosis” of NAFLD among 47.6% of the participants (false positives). At 95% sensitivity, our model showed 33.8% specificity, with 14.8% false negatives and 38.8% false positives. In turn, a specificity of 95% was associated with a sensitivity of 53.3% (35.0% false positives and 7.0% false negatives). According to the Youden statistic, the optimal cut-point corresponding to the maximum sum of sensitivity and specificity in the validated model was at 0.36 (sensitivity: 69.3%, specificity: 85.3%). Of note, none of the tested biomarkers of metabolism and inflammation (adiponectin, leptin, resistin, C-reactive protein, TNF-α, IL-6, IL-8 and IFN-γ) was significantly associated with liver fat content or NAFLD in our regression analyses. The application of previously published indices in our study sample revealed areas under the ROC curve of 0.82 [*NAFLD Liver Fat Score* by Kotronen et al. [9]], 0.74 [*FSI* by Long et al. [10]], 0.71 [*HSI* by Lee et al. [11]], and 0.77 [*FLI* by Bedogni et al. [12]] (see Table 4).

**Table 4:** Areas under the receiver operator characteristic curves (AUROC) for the prediction of NAFLD derived from different models in the HELENA-Trial (n = 143)

	Included Predictors	Reference	AUROC
NAFLD liver fat score	Metabolic syndrome, Diabetes, AST, AST/ALT ratio, Insulin	Kotronen et al. 2009 [9]	0.82 (0.75–0.89)
Framingham Steatosis Index (FSI)	Age, Sex, BMI, Hypertension, Diabetes, ALT/AST ratio, Triglycerides	Long et al. 2016 [10]	0.74 (0.65–0.82)
The Hepatic Steatosis Index (HSI)	Sex, BMI, Diabetes, ALT/AST ratio	Lee et al. 2009 [11]	0.71 (0.63–0.80)
The Fatty Liver Index (FLI)	BMI, Waist circumference, GGT, Trygylcerides	Bedogni et al. 2006 [12]	0.77 (0.70–0.85)
Helena-Trial Index	Age, Sex, Waist circumference, ALT, HbA1c, HOMA-IR	Present Study	0.85 (0.78, 0.91)^a^

^a^after internal bootstrap validation

Using an alternative cut-point for NAFLD at 5% liver fat instead of 5.56%, as proposed by some experts [18], in sensitivity analyses yielded highly similar results, as only two individuals with liver fat values between 5 and 5.56% had to be re-classified. The areas under the ROC curves with our prediction formula were 0.88 (95% CI: 0.82, 0.93) before and 0.84 (95% CI: 0.78, 0.91) after internal bootstrap validation. When we applied our formula to predict NAFLD among overweight individuals only, the area under the ROC curve upon bootstrap validation (0.80, 95% CI: 0.68, 0.92) was slightly lower compared than the one obtained among obese individuals only (0.84, 95% CI: 0.76, 0.93).

## Discussion

In the present study, we assessed anthropometric parameters and blood biomarkers as potential predictors of liver fat content and NAFLD among overweight and obese non-diabetics. Liver fat showed the strongest correlations with waist circumference (ρ = 0.52), ALT (ρ = 0.56), and insulin (ρ = 0.55) as well as HOMA-IR (ρ = 0.56). A multivariable linear regression model based on waist, ALT, GGT, HbA1c, insulin, and creatinine explained 54% of the variance in liver fat content. A similar combination of markers (age, sex, waist circumference, ALT, HbA1c, and HOMA-IR) showed a good prediction capacity regarding NAFLD, with an AUROC of 0.87, which was slightly attenuated after internal bootstrap validation (0.85).

Several groups have established indices based on anthropometry and routine biomarkers for the prediction of fatty liver disease before us, to our knowledge without external validation [9–12]; thus, one of our goals was to assess how existing indices perform in our study population. The *NAFLD Liver Fat Score* developed by Kotronen et al. [9], which is based on the presence of the metabolic syndrome and type 2 diabetes (dichotomous variables) in addition to insulin, AST, and the AST/ALT ratio, predicted NAFLD with an AUROC of 0.82 in our study population, even though individuals with diabetes were excluded from our study. This is consistent with our observation that the strongest predictors of NAFLD are markers of liver function (AST/ALT or ALT) and insulin sensitivity (insulin or HOMA-IR). Unlike in our prediction model, waist circumference was not part of the *NAFLD Liver Fat Score*. However, considering that waist circumference is a hallmark of the metabolic syndrome and type 2 diabetes [19], our regression model and the *NAFLD Liver Fat Score* contain highly similar sets of predictors [9]*.*

Our prediction models for liver fat on the continuous scale revealed a model R^2^ of 53.9% when using our own set of predictors (waist, ALT, GGT, HbA1c, insulin, and creatinine) vs. 41.4% when using the predictors selected by Kotronen et al. (AST, AST/ALT ratio, diabetes type 2, metabolic syndrome, and insulin) [9]. This difference may be due to the fact that we test a broader set of predictors for our model than Kotronen and colleagues, who obtained an adjusted R^2^ of 49% in their own study. In any case, it should be noted that either model explains only up to ~ 50% of the variance in liver fat so that both models may only facilitate a rough projection of true liver fat content [9].

The lack of parameters of insulin sensitivity in other previously published indices, i.e. *FLI, FSI,* and *HSI* [10–12] may explain why these revealed AUROCs below 0.8 in our sample. Interestingly, Bedogni et al., who developed the highly cited *FLI*, did find insulin levels to be a strong predictor of fatty liver in their study, but decided not to include insulin into the *FLI*, since it was not routinely measured in clinical practice [12]. The methodologies used for NAFLD assessment constitute another major difference between previous studies and ours. While Kotronen et al. used MRI-techniques (spectroscopy) for quantification of liver fat content, which is comparable with our MRI-technique [16, 20, 21], in the other studies ultrasound or computed tomography were used for assessing liver fat content, which are less precise compared to MRI-techniques [10–12, 22, 23].

Other than expected, none of the metabolic (adiponectin, resistin, leptin) and inflammatory factors (CRP, TNF-α, IL-6, IL-8 and IFN-γ), which have been proposed as potential circulating markers of NAFLD [24, 25], were significantly associated with liver fat content (%) and NAFLD in our study. Our analyses did not show associations for markers of iron status, either, although iron overload has been described as a feature of NAFLD [18, 26]. In part, the observed lack of associations with these blood-based biomarkers may be explained by the fact that our study population consisted of overweight and obese individuals who were free of diabetes and other metabolic complications, and that alterations of adipokine and cytokine signaling as well as iron metabolism may be related to the development of more advanced metabolic dysfunction. In this regard, it is important to note that most individuals in our trial had liver fat values below 30%.

Our study had several limitations. As stated above, we did not have the opportunity to validate our prediction model externally. Stratified analyses by different degrees of overweight and obesity were restricted due to the limited sample size, and out trial did not have a follow-up on major clinical outcomes. While our biomarker analyses covered a wide range of routine and putative novel markers of NAFLD, we did not have the opportunity to measure more costly non-routine parameters such as sCD36 or procollagen III N-terminal peptide, or to use commercial NAFLD tests based on circulating markers [24, 25, 27]. We did not have information on genetic markers of NAFLD [28]. However, PNPLA3 (patatin-like phospholipasedomain-containing 3), the only more established genetic marker of NAFLD, did not improve NAFLD prediction in the study by Kotronen et al. [9]. Finally, as in many other studies and routine clinical practice, we did not perform biopsies of the liver parenchyma, given the risk of complications so that no internal comparison between histological and MRI data was possible. Thus, and because the vast majority of our population had liver fat values below 30%, we could not assess different grades of steatosis. Nevertheless, MRI-techniques are a proven exact method for liver fat content assessment and are superior to ultrasound derived diagnosis of steatosis [18].

## Conclusions

In summary, NAFLD was predicted by a combination of waist circumference, ALT, HbA1c, and HOMA-IR at an AUROC of 0.85 (after internal validation) among overweight and obese adults, free of manifest metabolic complications. This result appears highly promising with respect to clinical routine care considering that the used predictors are easily available without relevant health care expenditures. Considering that all other potential first-line screening tools have specific disadvantages related to costs, patient burden, and sensitivity [29–32], our prediction model may be an interesting tool to identify persons at risk of NAFLD-induced steatohepatitis, fibrosis and cirrhosis, who could be pre-screened for further diagnostic tests by sonography, imaging or liver biopsy. Nevertheless, it has to be acknowledged that external validation of our prediction model is needed, and that the previously existing NAFLD prediction algorithm that performed best in our collective showed an AUROC of 0.82, which is very reasonable but not excellent. It remains to be seen whether novel markers beyond those we tested in the present study may further increase sensitivity and specificity of NAFLD indices, so as to justify a wider use in first-line screening.

## Additional file

Additional file 1: Electronic Supplementary Material: **Figure S1.** Histogram of MRI-derived liver fat content (%) values. **Figure S2.** Spearman’s correlations (ρ) between liver fat, anthropometric parameters and biomarkers. **Table S1.** Associations between individual predictors and the odds ratio of non-alcoholic fatty liver disease, sorted by decreasing area under the receiver operator characteristic curve (AUROC)*. (DOCX 123 kb)

## Abbreviations

ALT: Alanine transaminase
AST: Aspartate transaminase
AUROC: Area under the receiver operating characteristic curve
BMI: Body mass index
CRP: C-reactive protein
CVD: Cardiovascular disease
ECLIA: Electrochemiluminescence immunoassays
GRE: Gradient echo
HCC: Hepatocellular carcinoma
IFN-γ: Interferon-γ
IL-6: Interleukin-6
IL-8: Interleukin-8
MR: Magnetic resonance
MRI: Magnetic resonance images
MSD: Meso Scale Discoveries
NAFLD: Non-alcoholic fatty liver disease
NASH: Non-alcoholic steatohepatitis
PDFF: Proton density fat fraction
PNPLA3: Patatin-like phospholipasedomain-containing 3
ROI: Regions of interest
TNF-α: Tumor necrosis factor-α
TSH: Thyroid-stimulating hormone
