# Supplementary material for: Anthropometric and blood parameters for the prediction of NAFLD among overweight and obese adults
Source: BMC Gastroenterol. 2018 Jul 13;18:113. doi: 10.1186/s12876-018-0840-9 (PMC6045848; doi:10.1186/s12876-018-0840-9)
Supplement: Supplementary file 1 — Electronic Supplementary Material: Figure S1. Histogram of MRI-derived liver fat content (%) values. Figure S2. Spearman’s correlations (ρ) between liver fat, anthropometric parameters and biomarkers. Table S1. Associations between individual predictors and the odds ratio of non-alcoholic fatty liver disease, sorted by decreasing area under the receiver operator characteristic curve (AUROC)*. (DOCX 123 kb) [file 12876_2018_840_MOESM1_ESM.docx]

**Electronic Supplementary Material**

**Anthropometric and blood parameters for the prediction of NAFLD among overweight and obese adults**

Tilman Kühn^a^, Tobias Nonnenmacher^b^, Disorn Sookthai^a^, Ruth Schübel^a^, Daniel Antonio Quintana Pacheco^a^, Oyunbileg von Stackelberg^b^, Mirja E. Graf^a^, Theron Johnson^a^, Christopher L. Schlett^b^, Romy Kirsten^c, d^, Cornelia M. Ulrich^e, f^, Rudolf Kaaks^a^, Hans-Ulrich Kauczor^b^ and Johanna Nattenmüller^b*^

^a^German Cancer Research Center (DKFZ), Division of Cancer Epidemiology, Im Neuenheimer Feld 581, 69120 Heidelberg, Germany

^b^Heidelberg University Hospital, Diagnostic and Interventional Radiology, Im Neuenheimer Feld 110, 69120 Heidelberg, Germany

^c^National Center for Tumor Diseases (NCT), Liquid Biobank, Im Neuenheimer Feld 460, 69120 Heidelberg, Germany

^d^Division of Preventive Oncology, German Cancer Research Center (DKFZ) and National Center for Tumor Diseases (NCT), Im Neuenheimer Feld 460, 69120 Heidelberg, Germany

^e^Department of Population Health Sciences, University of Utah, 2000 Circle of Hope, Salt Lake City, Utah 84112-5550, USA

^f^Huntsman Cancer Institute, Salt Lake City, 2000 Circle of Hope, Salt Lake City, Utah 84112-5550,USA

***Corresponding author:**

Johanna Nattenmüller

University Hospital Heidelberg

Department of Diagnostic and Interventional Radiology

Im Neuenheimer Feld 110

D-69120 Heidelberg, Germany

Email: Johanna.nattenmueller@med.uni-heidelberg.de

Phone: +49 6221 5636462

Fax: +49 6221 56 573

**Figure S1** Histogram of MRI-derived liver fat content (%) values


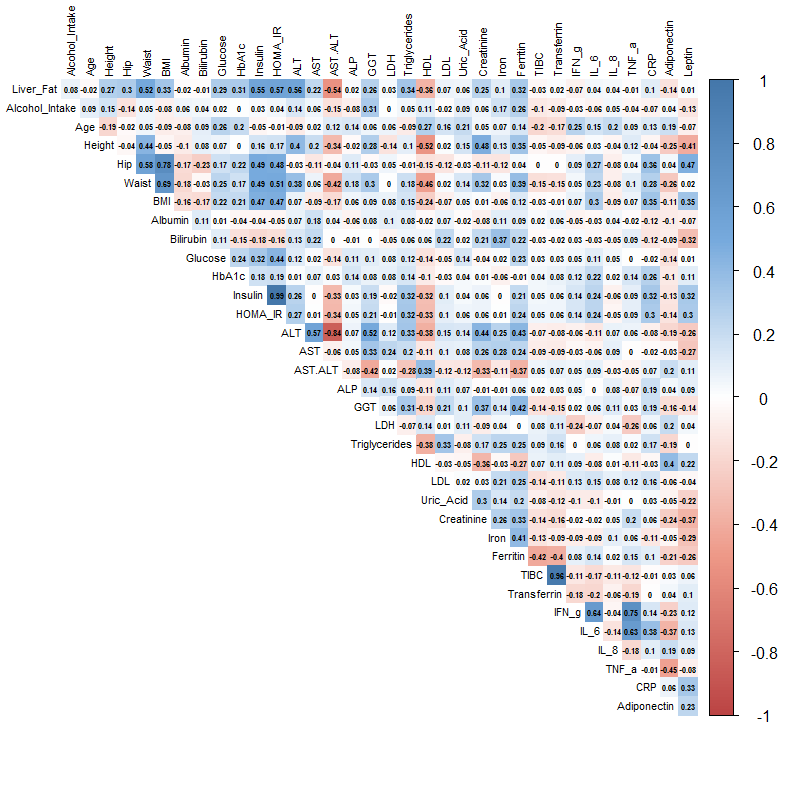


**Figure S2** Spearman’s correlations (ρ) between liver fat, anthropometric parameters and biomarkers

| **Table S1** Associations between individual predictors and the odds ratio of non-alcoholic fatty liver disease, sorted by decreasing area under the receiver operator characteristic curve (AUROC)* | | | | |
| --- | --- | --- | --- | --- |
|  | ***Odds Ratio*** | ***(95 % CI)*** | ***AUROC*** | ***(95 % CI)*** |
| ALT | 1.12 | (1.07, 1.18) | 0.78 | (0.70, 0.86) |
| Homa-IR | 3.32 | (1.91, 5.78) | 0.76 | (0.69, 0.84) |
| Insulin | 1.33 | (1.17, 1.53) | 0.76 | (0.68, 0.84) |
| Waist | 1.10 | (1.05, 1.14) | 0.75 | (0.67, 0.83) |
| AST/ALT Ratio | 0.11 | (0.03, 0.45) | 0.72 | (0.64, 0.81) |
| BMI | 1.20 | (1.08, 1.32) | 0.69 | (0.61, 0.78) |
| HDL | 0.96 | (0.93, 0.99) | 0.69 | (0.60, 0.77) |
| AST | 1.13 | (1.05, 1.22) | 0.69 | (0.60, 0.77) |
| HbA1c | 8.23 | (2.36, 28.7) | 0.68 | (0.60, 0.77) |
| Triglycerides | 1.01 | (1.00, 1.01) | 0.68 | (0.59, 0.77) |
| GGT | 1.04 | (1.01, 1.06) | 0.66 | (0.57, 0.75) |
| Glucose | 3.43 | (1.38, 8.47) | 0.66 | (0.57, 0.75) |
| Ferritin | 1.00 | (1.00, 1.01) | 0.64 | (0.55, 0.73) |
| Creatinine | 1.03 | (1.00, 1.07) | 0.63 | (0.54, 0.73) |
| Height | 1.05 | (1.00, 1.11) | 0.63 | (0.54, 0.72) |
| Leptin | 1.02 | (0.98, 1.06) | 0.62 | (0.53, 0.71) |
| Transferrin | 1.56 | (0.68, 3.62) | 0.61 | (0.52, 0.71) |
| CRP | 1.06 | (0.95, 1.17) | 0.61 | (0.52, 0.70) |
| TIBC | 1.02 | (0.97, 1.06) | 0.61 | (0.51, 0.70) |
| Bilirubin | 0.69 | (0.23, 2.09) | 0.61 | (0.51, 0.70) |
| LDH | 1.00 | (0.99, 1.01) | 0.61 | (0.51, 0.70) |
| Albumin | 0.96 | (0.83, 1.11) | 0.61 | (0.51, 0.70) |
| Adiponectin | 0.99 | (0.96, 1.03) | 0.60 | (0.51, 0.70) |
| TNF-alpha | 0.94 | (0.82, 1.07) | 0.60 | (0.51, 0.69) |
| LDL | 1.00 | (0.99, 1.02) | 0.60 | (0.50, 0.69) |
| Iron | 0.99 | (0.94, 1.04) | 0.60 | (0.50, 0.69) |
| Alk. Phosphatase | 1.00 | (0.98, 1.02) | 0.59 | (0.50, 0.69) |
| Resistin | 1.04 | (0.86, 1.26) | 0.59 | (0.50, 0.69) |
| Interferron-gamma | 1.00 | (0.98, 1.02) | 0.59 | (0.50, 0.68) |
| Interleukin-6 | 0.93 | (0.78, 1.10) | 0.59 | (0.50, 0.68) |
| Interleukin-8 | 0.99 | (0.95, 1.03) | 0.58 | (0.48, 0.67) |
| *Odds ratios (95 % confidence intervals) per unit increase in predictors from logistic regression models adjusted for age and sex; | | | | |
